# Symptom checkers: Gaps in their evidence base and suggestions for accuracy evaluation

**DOI:** 10.1177/20552076261487359

**Published:** 2026-09-29

**Authors:** Norbert Donner-Banzhoff, Helmut Schaefer, Nadine Schlicker, Annette Becker, Joerg Haasenritter, Veronika van der Wardt

**Affiliations:** 1 Department of Primary Care, 9377 University of Marburg, Marburg, Germany; 2 Coordination Centre for Clinical Trials, 9377 University of Marburg, Marburg, Germany; 3 Institute for Artificial Intelligence in Medicine, 9377 University Hospital Giessen Marburg & University of Marburg, Marburg, Germany

**Keywords:** MeSH, diagnosis, computers, handheld, research design, sample size, large language models, non-MeSH, diagnostic devices, symptom checkers

## Abstract

Symptom checkers (SCs) are mobile healthcare applications for the general population. Based on symptoms entered by the user, SCs provide a list of likely diagnoses and/or triage advice. Studies conducted so far show limited accuracy of current SCs with designs carrying a high risk of bias. In this narrative review we discuss three major methodological shortcomings of previous studies: 1) wrong study setting, 2) inappropriate reference standards, and 3) too small samples. To reflect the home setting, participants of an accuracy study must be prospectively recruited to document symptomatic episodes and health care used. Studies based on vignettes or samples drawn from healthcare facilities are not sufficient. A panel reviewing follow-up data must determine diagnoses for each episode and appropriate (non-)utilization of healthcare (delayed-type reference standard). Even when outcomes are collapsed into four groups, the sample size requirements for investigating triage appropriateness are considerable (n=62,000). The severity and treatability of rare conditions must be accommodated. Because of the defensive tendency of available SCs (with sensitivity maximized), the specificity will necessarily be limited to 20 to 30% at most, irrespective of the findings of an empirical study. We discuss the prospects for technological improvements, such as Large Language Models. For investigations of their accuracy, the same design requirements apply as for established models. Accuracy evaluation should be guided by the *function* of the intervention under consideration independently of the technology used. Given the limitations of our knowledge regarding SCs’ accuracy, their place in the health care system is far from clear.

## Introduction

Symptom checkers (SC) have gained worldwide popularity, especially for the young, female and well educated.^1^ They provide diagnostic assessment to laypersons independent of the professional healthcare system. Users enter their symptoms into a digital, often mobile, device. As a result, they obtain a list of diagnoses and their probabilities, as well as advice regarding what to do (triage).^2^ In the following we discuss digital devices to be used in the home setting by laypersons, which are not restricted to a particular topic (symptom, disease etc.). We also exclude applications intended to monitor, motivate, or serve other purposes than diagnosis.

Although developers caution that their devices are not meant to replace doctors, they appear attractive: providing quick access to plausible advice, satisfying the user’s curiosity, and coming with special authority or even enchantment.^3^ From a healthcare systems perspective, they promise to reduce pressure on emergency services so that their use is officially encouraged within several healthcare systems.^4^

One core question relevant to this technology refers to the accuracy of SCs: are diagnoses provided by the device valid? Is triage advice justified? Several scoping^2,5,6^ and systematic^7–11^ reviews (SRs) have addressed this question (we included only reviews on generally applicable tools, published in English language). Authors of SRs without exception have found grave deficits in both triage and diagnosis. Moreover, study designs and their execution suffered serious limitations, such as using vignettes (case descriptions with assumed true diagnosis) for evaluation or too small samples of unclear representativeness, lack of justification (formal calculation of required size), data input by clinicians (instead of users themselves) to name but a few examples. Lastly, triage advice from SCs is generally risk averse, encouraging users to seek care for conditions where self-care is reasonable.^9^

The objective of this narrative review is to provide a critique of research designs currently employed to investigate the accuracy of SCs, and to develop recommendations for improvement. To this end, we provide an in-depth discussion of the limitations of the evidence base of currently available SCs. We discuss these in turn with suggestions for improvement. We conclude by discussing novel developments and prospects for evaluation.

## Accuracy: Limitations of current research

In our view, there are three major requirements for the investigation of diagnostic interventions which are important, but which are not fulfilled by current research of SCs and their accuracy:

− study setting not appropriate
− reference standard not adequate
− study sample too small

Individual studies contributing to systematic reviews were independently assessed by two independent researchers. If a systematic review including supplemental material did not provide the required information, the individual paper was obtained and assessed. Disagreements were discussed and clarified (see supplemental material A [columns] for criteria of interest).

### Inappropriate setting

It is widely acknowledged in diagnostic research that studies must be conducted in the settings where decisions are made they are supposed to inform.^12^ Not only does disease prevalence increase from primary to secondary care (and presumably from lay to primary care), but the accuracy of signs may also differ because their presence may have led to (self-)referral. Samples drawn from different settings thus have their specific characteristics limiting generalization from one to another. SCs are targeted at the home setting, i.e. persons (strictly speaking they are not [yet] patients) having noticed symptoms, who wonder what these mean and whether they should seek professional help. Evaluating SCs with patients attending a health care facility, such as an emergency unit, is thus of limited value, because these participants have already decided that their problem requires professional care. Individuals not asking for professional help are not represented in such samples. Studies restricted to a particular symptom, organ or group of diseases^13^ are not helpful either because by their design they imply a selection which any individual pondering on symptoms and potential sources of care must make.

Case vignettes are problematic for the evaluation of SCs, because they tend to reflect investigators’ intuitions and ideas. Real cases are less clear-cut, only vaguely demarcated from each other and often defy diagnostic categorization^14^ (chapters 1.13, 5.5, 6).^15^ This applies to primary care, and presumably even more so to the home setting.

Two thirds of studies included in the SRs mentioned in the introduction were based on vignettes. Only two drew participants from the general population without restriction to certain clinical topics^16,17^ (for details see supplemental material A, Table).

**Recommendation**: For a valid study of SCs, participants have to be recruited prospectively and be asked to record symptomatic episodes during a defined follow-up period, even if these do not lead to professional consultation.

### Inadequate reference standard

Of individual studies which were not based on vignettes and not restricted to a particular topic (n=9), seven recruited participants from healthcare facilities and retrieved reference diagnoses from patient records. This is convenient but allows an evaluation only for participants who seek professional help. Only one study included by SRs seemed to have used a physician panel, although details of the procedures are not given.^18^ We had to exclude this study from our survey, because the publication (company homepage) is not available anymore. No study included in SRs provided an investigation of all participants or at least a random subset of those triaged to low priority (for details see supplemental material A, Table).

In studies evaluating diagnostic tests, the performance of the index test is compared to a reference standard. Given the broad range of health problems SCs are expected to deal with, a panel of clinicians is required to determine the pathology underlying symptoms and findings.^19^ After a specified time period the panel can review relevant clinical information to establish a reference diagnosis.^20^ Moreover, panels can evaluate triage recommendation by deciding which action would have been appropriate in retrospect. This “delayed-type reference standard”, however, requires follow-up of patients by phone or other means to establish the course of symptoms, resulting treatments, to obtain discharge letters etc. Outcome assessment based on health care records alone will only be an option in a health care system with complete documentation of all contacts. But again, the course of symptomatic episodes without professional care would not be captured.

**Recommendation**: After an illness episode with use of an SC, study participants must be followed up to obtain information on course of illness and treatments. This information must be reviewed by a panel of clinicians to establish a reference diagnosis and adequate triage.

### Too small samples

Although two studies recruiting from the general population included 3812^16^ and 4456^17^ participants respectively, in each less than 200 were investigated regarding their reference diagnoses or need for care (for details see Supplementary Table 1). Were these samples sufficiently large to estimate SCs’ accuracy?

The prevalence of serious diseases varies between settings. In primary care, their prevalence is low, in the home setting presumably even lower.^21^ However, it is precisely for this group of diseases where accurate recognition and subsequent treatment matters. For the far more common minor symptomatic episodes, wait-and-see and/or alleviation of symptoms are appropriate. A precise diagnosis is not warranted in these cases. A potential for harm lies with much less frequent, specific diseases, where timely treatment makes a difference for the well-being and prognosis of the patient.

For estimating accuracy regarding less frequent, specific conditions many more subjects are needed than in all studies published so far, where this distinction has not been made. We calculated the sample size requirement for a study investigating triage recommendations. As the coarsest granularity^22^ we suggest the following:

i. self-limiting states, no or only symptomatic treatment possible, e.g. non-specific headache, episode of vomiting and diarrhea
ii. benign or mild disease, specific treatment available, e.g. acute urinary tract infection
iii. serious subacute or chronic disease, specific treatment to improve prognosis and/or quality of life available, e.g. malignancy, rheumatoid arthritis, Parkinson’s disease
iv. acute, life-threatening illness, e.g. intestinal obstruction, ectopic pregnancy, acute myocardial infarction.

Although each of these broad categories include heterogenous diseases, they have common implications regarding the urgency and availability of treatments.

We assume that 0.5 % of problems motivating the use of an SC belong to category iv), categories i)-iii) being more common (for derivation and justification as well as sensitivity analyses regarding different prevalences of category iv see supplemental material B). Assuming a probability of a correct classification of 0.9 in each of the four categories, which we want to be estimated with a precision of +/- 0.05 each, we need a sample of 310 episodes in each of the four categories. With n=310, the 100%·γ Clopper-Pearson confidence interval would be - 0.05 to + 0.038 for a point estimate of 0.9, where γ was set to $\sqrt[{4}]{.95}$ to obtain simultaneous confidence intervals for 4 groups i to iv (Šidák). In order to have enough cases in the least frequent category iv), we would have to include overall 62,000 episodes in the study (see supplemental material B for details, including a sensitivity analysis of different prevalences of category iv). If individual participants are allowed to contribute more than one episode, the resulting reduction of participants to be included is likely to be (partially) offset by adjustment for cluster effects. This calculation with possible scenarios reduced to four categories applies only to triage recommendations. For diagnostic suggestions, usually presented as a list with associated probabilities, much higher numbers of participants would be required.

**Recommendation**: Sample size must be calculated so that severe and urgent conditions, for which professional help is decisive, are accounted for with sufficient precision.

## Evidence for the accuracy of SCs: current state and recommendations

Studies limited to certain symptoms or diseases, those using case vignettes, or being conducted in health care settings, may provide interesting evidence, potentially contributing to product development and optimization (“bench testing”). Similar considerations apply to simulated or routine datasets. However, they do not provide confirmatory evidence for the accuracy of SCs in analogy to phase III trials for drugs (for the distinction between exploratory and confirmatory research in healthcare see Ref. 23; although written with biomedical innovation in mind, research questions and designs apply to our topic). Of the studies included in five SRs, only two were non-vignette, not restricted to a particular topic and recruiting participants outside healthcare facilities.^16,17^ These two, however, suffered from insufficient reference standard (questionnaire or standardized phone call by nurse for only a subset of participants) and sample size (see supplemental material A [Table]).

Although SCs are not classical diagnostic tests, the design features proposed here have been derived from cross-sectional diagnostic studies.^24^ Study designs can be adapted to the low-prevalence setting^25^ and to more than one disease outcome.^26^ However, given the multiplicity of outcomes (=diseases of interest) the possibilities to reduce the expense for an evaluation of SCs are limited. Respective frameworks for publication,^27^ health informatics^28^ device regulation^29^ and AI-based medical device software,^30^ unfortunately, lack specificity regarding research designs for clinical evaluation.

## Risk avoidance of current algorithms

Only very rarely do we encounter pathognomonic symptoms or findings, i.e. those which point consistently and exclusively to a particular disease. Most are ambiguous in that they allow hypotheses regarding underlying pathological states only in combination. Even then conclusions are fraught with uncertainty.^31^

Current SCs are risk averse. The proportion of episodes in which a medical consultation is being proposed is as high as 85%^16^ or 86%.^32^ Among 23 devices investigated by Semigran et al., four suggested professional help in all cases by their design.^7^

Developers of SCs face a dilemma. If they set thresholds for a recommendation to contact a professional provider high, they encourage self-help and relieve pressure on acute services. They may, however, cause harm by users forgoing effective care for treatable conditions. At present, they prefer to err on the safe side by setting thresholds for professional care very low, in other words suggesting consultation for most complaints. Although this strategy avoids negative publicity and legal consequences, it comes at a cost by limiting the specificity of devices, as the following calculation shows.

By collapsing the strata of possible triage advice into two (self-treatment vs. professional consultation), one can illustrate the effect of a risk averse approach. With a device suggesting professional help in 85% of episodes,^16,32^ a sensitivity of 90% and a prevalence of professional consultation being justified according to the reference panel of 20%, the specificity of the device is as low as 16%. Assuming a very high prevalence of 50% and a sensitivity of 95% or even 100%, the specificity is 21.5% or 30%, respectively. These estimates may be too optimistic, because we do not know whether sensitivities as high as that can be achieved by current SCs (for sensitivity analyses see supplemental material B).

We do not know how the risk averse tendency affects users’ trust in the device and subsequent behavior. How do users react once they realize that they are spared a professional consultation only in rare cases? Do they follow the suggestion given by the device? Do they ignore triage advice, as surveys^33^ and a discussion with our patient participation panel have suggested, and are primarily interested in differential diagnoses provided, not least for their entertainment value? Are health anxiety and uncertainty alleviated or increased by SC use (anxious individuals are apparently more likely to use SCs^34^)?

## Future developments

### Novel technologies

Improving the accuracy of SCs would be a way out of the risk-uncertainty-conundrum. While current SCs are based on probabilistic symbolic algorithms, machine learning is increasingly used for medical applications. The diagnostic task addressed by SCs, however, differs from, for example, the processing of medical images. While the latter produce outputs of limited complexity, usually something like “condition/finding present YES/NO”, the former would have to consider hundreds of possible outcomes (=diagnoses). The sheer number of degrees of freedom makes the clinical diagnostic setting unique. An extremely high number of cases would be required for learning with the inputs specified, recorded, coded, and, for supervised learning, reference diagnoses provided by an expert panel (these would be required also for confirmatory evaluation of nonsupervised learners). The expense for such an endeavor would be very high.

Devices based on large language models (LLM), such as ChatGPT or PaLM, are promising in answering questions concerning *general* medical knowledge,^35^ although their success seems to vary.^36^ The task of SCs and similar devices, however, is different: They are supposed to tell the user about the state of her body (which in this case includes her mental state). Their application for triage^37^ and diagnosis is currently being investigated. In a vignette study, a consumer health tool frequently overtriaged nonurgent cases (35%) and undertriaged emergencies (48%).^38^ A LLM originating from a simulated learning environment has shown promising performance in a vignette study modeled after objective structured clinical examinations (OSCE). Here the model even showed higher diagnostic accuracy and superior performance than primary care physicians.^39^ Whether this model trained on specific diseases occurring in specialized care performs accurately in the setting where SCs are currently employed, remains to be seen.

According to a recent scoping review,^40^ LLM-based diagnostic systems developed so far focus on single fields or issues, instead of the general remit of SCs. Developers suggest assisting clinicians in their diagnostic work as the purpose of their products (^40^ page 8) or devices as auxiliary tools.^41^ This differs from SCs’ original purpose of advising individuals before entering the healthcare system.

Although LLM-based diagnostic systems make a free dialogue between user and device possible, this leads to additional vulnerabilities. If the model is not presented the full case but data collection is left to the interaction between user and device, accuracy deteriorates rapidly,^42^ especially with untrained operators.^43^ Apparently, users fail to communicate information essential for diagnosis. The advantage of statistical actuarial methods compared to human decision-making,^44^ i.e. their consistency, is diminished or even lost with LLMs.

In comparison to human clinicians, digital diagnostic devices are confined to a more limited set of data. Clinicians have no restriction in asking additional questions, they notice how symptoms are being described, they have a visual impression of the state of the patient and can perform a physical examination according to diagnostic hypotheses entertained. In settings with continuity of care, they can even compare current symptoms and findings to previous illness episodes. They are aware of the physical and social context the patient comes from. In other words, the clinical consultation is a “high-context” setting whereas current digital devices are limited to low-context information.^45^

As long as they work with the same clinical information as established SCs (or even less, see Ref. 43), advanced digital technologies can only finetune their model of associations between the variables contained in the dataset available to them. Dramatic improvements regarding their accuracy based on improved models alone are unlikely to result. Finally, novel devices based on LLMs or other technologies will have to comply with the standards outlined in this paper.

### Prospects for evaluation

Ten years ago, Wyatt deplored the lack of evidence regarding the accuracy of digital diagnostic devices.^46^ Measured against the standard of the study design outlined in this paper, the situation has not substantially changed, nor does current, admittedly insufficient evidence suggest that SCs were getting more accurate.^47,48^

Not enough thought is given to valid evaluation, or they are even placed outside the scope of conventional science.^49,50^ That for a recent statement on the regulatory evaluation of AI-based medical device software no clinical epidemiologist or biostatistician was involved, is disconcerting.^30^

Given the sample size required, the cost for a valid study of SCs’ accuracy will be very high. Given devices’ risk aversion resulting in low specificity, a funding body might quite understandably ask whether such a study is worthwhile. However, if we regard digital support for people with symptoms as important, we need reliable evidence regarding their accuracy.

Similar consideration as for SCs apply to other digital diagnostic tools, such as triage services, where non-physician call-handlers question patients and feed the information into a computer to obtain advice on how to proceed. Well known examples are the NHS111 system in the UK or the Dutch Triage System in the Netherlands.^51^

Currently SCs are classified as low-risk medical devices.^29,52^ It has been suggested that allocating SCs to a higher risk-class by regulatory bodies might lead to more systematic post-marketing surveillance (PMS).^9^ PMS taken seriously, however, would face considerable difficulties, if more than occasional anecdotes reaching sponsors were to be considered. Since SCs are used outside the professional healthcare system, systematic feedback and analysis of events (causal ascriptions) would be difficult to obtain and costly. To establish a denominator for PMS, users would have to be registered prospectively, which requires an effort similar to the confirmatory study design outlined above.

A high-risk classification would require more pre-approval evidence than currently available. The current EU-Directive^29^ to mention one example, requires clinical investigation, i.e. explicitly conducted clinical trials, to be conducted for conformity assessment for high-risk devices (Article 61 no 4). Annex XV Chapter 1 specifies “an adequate number of observations to guarantee the scientific validity of the conclusions” (no 2.1). Moreover, “The research methodologies used to perform the clinical investigation shall be appropriate to the device under investigation” (no 2.3). Investigations need “a clinical environment that is representative of the intended normal conditions of use of the device in the target patient population” (no 2.4). Lastly, “endpoints of the clinical investigation shall address the intended purpose, clinical benefits, performance and safety of the device. The endpoints shall be determined and assessed using scientifically valid methodologies. The primary endpoint shall be appropriate to the device and clinically relevant” (no 2.6). For the US, FDA regulations are more lenient. SCs are an area where the authority exercises “enforcement discretion”.^53,54^

The design features we have outlined here would satisfy EU-requirements. For devices based on LLMs, the Artificial Intelligence Act published by the EU in 2024 applies independently of national legislation. However, issues regarding the regulation of AI in general and medical devices in particular still have to be resolved.^55^

That SCs are used outside the professional healthcare system might justify their allocation to a higher risk category. For diagnostic devices being employed in the presence and under at least indirect supervision by professionals, such as with triage systems for making practice appointments, less stringent requirements seem appropriate.

## Conclusion

Digital solutions have inspired utopias of a better and easier life. In so far as these apply to healthcare, digital diagnostic systems are often proposed to replace human clinicians. In a more modest perspective, one could welcome SCs as a possibility for those who lack (timely) access to personal healthcare. However, the accuracy and trustworthiness of these solutions become an issue, once we expect them to become the “digital front door” of the health care system.

Societies, governments and professional communities require drugs, medical devices and systems of care to be evaluated for their effectiveness and safety to avoid unnecessary expense and harm to the population. Methods have been developed to formulate research questions and objectives, in our case accuracy as the most important, also suggesting study designs, study procedures and statistical analysis. Only after investigations of this kind have been concluded, do we know whether a claim is justified or not. The systematic reviews discussed in this paper differ regarding their methods, search strategies and retrieved samples. They agree, however, that the accuracy of current SCs is deficient, as are study designs used for evaluation.

We suggest that digital solutions in the health field should not be exempt from the requirement of reliable evidence before being recommended and used. Evaluation should be guided by the *function* of the intervention under consideration independently of the technology used, in other words: novel technologies for SCs do not invalidate the methodological requirements specified in our paper.

## Supplemental material

Supplemental material - Symptom checkers: Gaps in their evidence base and suggestions for accuracy evaluation Supplemental material for Symptom checkers: Gaps in their evidence base and suggestions for accuracy evaluation by Norbert Donner-Banzhoff, Helmut Schaefer, Nadine Schlicker, Annette Becker, Joerg Haasenritter, Veronika van der Wardt in DIGITAL HEALTH

Supplemental material - Symptom checkers: Gaps in their evidence base and suggestions for accuracy evaluation Supplemental material for Symptom checkers: Gaps in their evidence base and suggestions for accuracy evaluation by Norbert Donner-Banzhoff, Helmut Schaefer, Nadine Schlicker, Annette Becker, Joerg Haasenritter, Veronika van der Wardt in DIGITAL HEALTH
