# Supplementary material for: Symptom checkers: Gaps in their evidence base and suggestions for accuracy evaluation
Source: Digit Health. 2026 Sep 29;12:20552076261487359. doi: 10.1177/20552076261487359 (PMC13769915; doi:10.1177/20552076261487359)
Supplement: Supplementary file 1 [file sj-pdf-1-dhj-10.1177_20552076261487359.pdf]

Norbert Donner-Banzhoff et al. Symptom checkers: Gaps in their evidence base and suggestions for the accuracy evaluation of digital diagnostic devices

Supplemental material A: Relevant studies extracted from published systematic reviews

Of studies included in published systematic reviews we have **successively** excluded vignette studies (col 3), and those addressing a restricted topic (col 4 - certain symptoms, diseases, or groups of diseases). Of the remaining (col 5) excluded those recruiting participants from a healthcare facility (col 6). Of the studies then remaining (humans as participants, no restriction to clinical topic, drawn from general population) we show sample size (col 7), study objective (col 8), endpoint evaluation (col 9), whether formal sample size calculation was presented (col 10), and reference (publication) (col 11).

For references see 'Reference' Section of main manuscript.

We identified one additional study which would have been eligible to be included in systematic reviews but was published after the period covered by the latest review (Riboli-Sasco et al. 2023):

> Cotte F, Mueller T, Gilbert S, Blümke B, Multmeier J, Hirsch MC et al. Safety of Triage Self-assessment Using a Symptom Assessment App for Walk-in Patients in the Emergency Care Setting: Observational Prospective Cross-sectional Study. JMIR Mhealth Uhealth 2022; 10(3):e32340

For this single-centre study patients were recruited from an academic medical centre emergency department. If included in the Table below, the study would have appeared in column 6 ("recruited from healthcare facility")

[…]

1	2	3	4	5	6	7	8	9	10	11
Publication systematic review	Number of studies in systematic review	Vignette studies (number)	Restricted topic of non-vignette studies (number)	Studies with Human participants AND general (number)	Of studies in column 5: recruitment from healthcare facility	Sample size (range), only 5 minus 6)	Study objective	Endpoint evaluation	Sample size calculation	Individual publication

Semigran et al. 2015	23	23								
Chambers et al. 2019	16 (SC as self-contained intervention) [1]	6	7	3	2	3812 completed triage and received advice, 192 investigated	Use of and compliance with triage system	Questionnaire on compliance with advice	No	Nijland et al.: 2010
Wallace et al. 2022	10 (for triage and diagnosis combined)	5	3	2	2					
Gottliebsen et al. 2020	Mostly opinion papers, narrative reviews, implementation studies, utilization studies without outcome evaluations. Only 4 regarded as contributing to accuracy of 17 overall<br>Niijland et al.	4		0						

[1] Babylon Health 2017 excluded because report by company website not available anymore. Company shut down because of financial difficulties and intervention by regulator.
[…]

	mentioned as implementation study									
Riboli-Sasco et al. 2023	14 (for triage and diagnosis combined)	7	1	6	5	4456 participants in survey, 126 investigated	Triage accuracy	Standardized phone call by nurse	No	Verzantvoort et al. 2018

Supplementary Table: Systematic Reviews of the accuracy of symptom checkers (SC) – design features of individual studies
