# Supplementary material for: Symptom checkers: Gaps in their evidence base and suggestions for accuracy evaluation
Source: Digit Health. 2026 Sep 29;12:20552076261487359. doi: 10.1177/20552076261487359 (PMC13769915; doi:10.1177/20552076261487359)
Supplement: Supplementary file 2 [file sj-pdf-2-dhj-10.1177_20552076261487359.pdf]

Norbert Donner-Banzhoff et al. Symptom checkers: Gaps in their evidence base and suggestions for the accuracy evaluation of digital diagnostic devices

Supplemental material B: Sample size considerations and sensitivity analyses

Derivation and justification of sample size

In the following we focus on the disease categories i to iv as specified in the main text and related triage advice given by SCs. Studies investigating the "Ecology of Care" (Green et al. 2001 – see references in main text) suggest that the prevalence of diseases grouped at four levels (see above) decreases from i to iv. According to Green et al., 80% of citizens surveyed had at least one symptom (=episode), 26.5% had medical (ambulatory) care, 1% of population were hospitalized within a month.

The unit of reporting was the individual ("at least one symptom/contact with health care system within a month"). While the numerator "hospitalization" can be assumed to be stable irrespective of the unit of reporting (an individual is unlikely to be hospitalized more than once within a month), the "number of symptomatic episodes" is likely to be larger than assumed by Green et al. (an individual often has more than one episode of symptoms within a month).

The study reported by Green et al. was descriptive, i.e. they did not judge the adequacy of care or levels of care obtained. The fact that an individual was admitted to hospital thus did not necessarily mean that she suffered serious illness but that a clinician assumed such illness to be sufficiently likely to justify admission, maybe even an urgent one. On the other hand, a large proportion of admissions presumably was elective for non-life-threatening disease. Considering the difference in denominators assumed between Green et al. and our topic as well as the ambivalence of their hospitalization rates we assume that in a prospective study 0.5% of episodes included would be associated with serious illness requiring urgent medical attention according to the panel's assessment (category iv). For a sensitivity analysis of different prevalences see below.

For sample size calculation we use a generalisation of the procedure described by Obuchowski (Obuchowski NA. Sample size calculations in studies of test accuracy. Stat Methods Med Res 1998; 7(4):371–92) in her section 3, paragraph 3. We are interested in the concordance of the triage advice provided by the SC and the "ground truth" in each of the four categories *i* to *iv*, in other words: the study should be large enough to simultaneously estimate all four concordance rates (i.e. the percentages of correct classification in each category) $r_i, r_{ii}, r_{iii}, and\ r_{iv}$. The required estimation precision, b, by which we mean the minimum distance of a concordance rate estimate to its upper or lower confidence limit, has to be selected as an input for the sample size determination. As the precision of a rate estimate depends on the unknown true value of the respective concordance rate r, we also have to select a value of interest, r_{int}. In other words: If in the accuracy study, the concordance rate in a subgroup is of the magnitude of the selected value r_{int}, the study should be able to estimate this rate with a precision defined by $r_{int} \pm b$. An approximate initial value for a required minimal sample size in every subgroup (category) may be obtained from the following formula (z = standard normal quantiles):

$$n = z^2_{(1+\gamma)/2} \cdot \frac{r_{int} \cdot (1 - r_{int})}{b^2},$$

where γ denotes the confidence level (see Table 2, Formula T2 in Obuchowski 1998). Since we want to achieve simultaneous confidence interval estimation in all four subgroups at an overall level of 0.95, we use $\gamma = \sqrt[4]{0.95}$. Starting with the approximate value from this formula, we calculated Clopper-Pearson confidence intervals under different choices of the sample size until we reached the required estimation precision, b . Assuming that the concordance rate was 0.9, to be estimated with a precision of 0.05, we start with n=223 from the formula

above and end up with *n*=310. With n=310, the Clopper-Pearson confidence interval would be - 0.05 to + 0.038 for a point estimate of 0.9, where $\sqrt[4]{0.95}$ was set to obtain simultaneous confidence intervals for 4 groups i to iv (Šidák). We require a sample of 310 episodes in every category as judged by the panel. Since we assumed that 0.5% of episodes to be category *i*, and a higher proportion of episodes in all other categories, overall 62,000 episodes would have to be recruited into the study.

Since we do not know the true value of the concordance rate, one should analyse the estimation precision which can be achieved with the selected subgroup sample size for a range of values of the unknown concordance rate. For example, with n=310, a rate of 0.5 (which is the most unfavourable case with respect to estimation precision) would be estimated with a precision of + 0.072 … - 0.072.

As soon as study participants can contribute more than one episode, the assumption of independence required for Clopper-Pearson confidence intervals does not hold any more. Additional episodes either would have to be excluded or adjustments for cluster effects be made, which would require more than 62,000 specified above.

Against the background of functions available with current SCs, our discussion of sample size has been limited to four categories and their related triage suggestions as only one, albeit important, outcome. Lists of possible differential diagnoses and their probabilities imply near-infinite degrees of freedom for statistical analyses. Study protocols thus require precise pre-specification of planned analyses including adequate α-adjustment for multiple testing or simultaneous confidence intervals to achieve a valid confirmatory statistical evaluation. If one wants to include further confirmatory statistical analysis in addition to concordance rates for triage, both the statistical analysis and the sample size planning has to be adapted to these extended confirmatory study objectives, which may considerably increase the resources needed to conduct the study.

Prevalences of category iv diseases – Sensitivity analysis

Prevalence of category iv episodes	Sample size required to capture 310 category iv episodes (=310/prevalence)
0.001	310,000
0.005	62,000
0.01	31,000
0.02	15,500

Specificity depending on sensitivity, consultation rate (recommendation) and prevalence

Comment: Please note that current SCs operate with recommended consultation rates as specified in the left columns. Only specificities provided in this column are realistic for current devices.

Sensitivity 0.99

Consultation rate	0.85	0.7	0.5
Prevalence			
0.2	0.185	0.373	0.623
0.4	0.243	0.493	0.827
0.6	0.360	0.735	NA

Sensitivity 0.9

Consultation rate	0.85	0.7	0.5
Prevalence			
0.2	0.163	0.350	0.600
0.4	0.183	0.433	0.767
0.6	0.225	0.600	NA

Sensitivity 0.8

Consultation rate	0.85	0.7	0.5
Prevalence			
0.2	0.138	0.325	0.575
0.4	0.117	0.367	0.700
0.6	0.075	0.450	0.950
